# Applying the InterVA-4 model to determine causes of death in rural Ethiopia

**DOI:** 10.3402/gha.v7.25550

**Published:** 2014-10-29

**Authors:** Berhe Weldearegawi, Yohannes Adama Melaku, Mark Spigt, Geert Jan Dinant

**Affiliations:** 1 Department of Public Health, College of Health Sciences, Mekelle University, Mekelle, Ethiopia; 2 INDEPTH Network, Accra, Ghana; 3 CAPHRI, School for Public Health and Primary Care, Maastricht University, Maastricht, The Netherlands

**Keywords:** InterVA, cause of death, Health and Demographic Surveillance System, chronic non-communicable, Ethiopia

## Abstract

**Background:**

In Ethiopia, most deaths take place at home and routine certification of cause of death by physicians is lacking. As a result, reliable cause of death (CoD) data are often not available. Recently, a computerized method for interpretation of verbal autopsy (VA) data, called InterVA, has been developed and used. It calculates the probability of a set of CoD given the presence of circumstances, signs, and symptoms reported during VA interviews. We applied the InterVA model to describe CoD in a rural population of Ethiopia.

**Objective:**

VA data for 436/599 (72.7%) deaths that occurred during 2010–2011 were included. InterVA-4 was used to interpret the VA data into probable cause of death. Cause-specific mortality fraction was used to describe frequency of occurrence of death from specific causes.

**Results:**

InterVA-4 was able to give likely cause(s) of death for 401/436 of the cases (92.0%). Overall, 35.0% of the total deaths were attributed to communicable diseases, and 30.7% to chronic non-communicable diseases. Tuberculosis (12.5%) and acute respiratory tract infections (10.4%) were the most frequent causes followed by neoplasms (9.6%) and diseases of circulatory system (7.2%).

**Conclusion:**

InterVA-4 can produce plausible estimates of the major public health problems that can guide public health interventions. We encourage further validation studies, in local settings, so that InterVA can be integrated into national health surveys.

Information about causes of death (CoD) is needed by health managers and policy makers at all levels of governance (1, 2). In countries where registration of vital events and medical documentation of death are practiced, such information can easily be generated. However, three quarters of the world's total population lives in countries where registration of vital events and CoD certification are not in place (3). Verbal autopsy (VA) is a technique growing in importance for estimating the CoD in populations without vital registration or other medical death certification and where the proportion of people who die at home is high (4, 5).

VA means that trained data collectors interview the caregivers or family of a recently deceased person, asking about signs and symptoms preceding the death, which are then interpreted into a probable cause of death. It is now widely used to estimate cause-specific deaths in research and for routine registration of deaths (1, 6, 7). Physician review, the commonly used method to derive a probable CoD from VA data, is a costly, slow, and non-reproducible process. Recently, a computerized method for interpretation of VA data (InterVA) has come into use. The InterVA process is comparatively fast and cheap and it is reproducible over time and place (7). It is also aligned with the WHO 2012 VA standard (8).

In Ethiopia, routine registration of vital events is non-existent and death certification is not compulsory. Thus, producing consistent, timely, and reliable CoD data has remained a challenge. Therefore, we used the InterVA method to interpret VA data from a rural population in Ethiopia.

## Methods

### Study setting

The Kilite Awlaelo Health and Demographic Surveillance System (KA-HDSS) is a longitudinal population-based surveillance site located about 802 km north of Addis Ababa, Ethiopia. The KA-HDSS is a member of the International Network for the Demographic Evaluation of Populations and Their Health (INDEPTH) Network [http://www.indepth-network.org/]. The KA-HDSS was started in September 2009 with a baseline population of 66,438 individuals living in 14,453 households. Data on household and vital individual events (pregnancy status, birth, death, marital status change, and migrations) and VA data were collected during house-to-house visits twice a year.

### VA questionnaire and interview

The VA questionnaire was adapted from the WHO, INDEPTH Network, Sample Vital Registration with Verbal Autopsy (8, 9). It has three separate questionnaires for the three age groups: neonate, post-neonate and children (29 days to 15 years), and adults (>15 years). Deaths were identified by data collectors during regular visits to the households. An adult relative of the deceased, who was the caregiver during the terminal illness, was interviewed by trained data collectors who completed at least high school. Data were collected after the end of the mourning period (45–55 days) using the paper format which on average takes 110 min to fill out.

### Interpretation of VA data

The InterVA-4 model (version 4.02) was used to interpret VA data into probable cause(s) of death. As described by Byass et al. (7), the model is based on Bayes’ theorem, which calculates the probability of a set of CoD given the presence of indicators (circumstances, signs, and symptoms) reported in VA interviews (7, 10). The InterVA requires extraction of a defined set of indicators from the VA questionnaire, and then processes these indicators to generate a summary of as many as three possible CoD with their corresponding likelihood (2, 11). Fractional causes are then aggregated and any residual component (where fractional causes total less than 1) ascribed as indeterminate. This approach thus integrates a measure of the individual uncertainty with which InterVA-4 is able to assign cause(s) of death into the analysis for each case. The InterVA model assigned indeterminate cause, either to a certain fraction of a single case (indeterminate) or as a whole (completely indeterminate). However, if the VA questionnaire did not contain usable data, it was excluded from the analysis.

Before interpretation of VA data to likely CoD, InterVA-4 requires labeling the incidence of malaria and HIV/AIDS in the study setting as ‘high’ or ‘low’. In Ethiopia, the prevalence of malaria and HIV/AIDS is 1% and 1.5%, respectively (12, 13). Thus, for the current report, levels for both Malaria and HIV/AIDS were set as “low”. The current report is based on VA for deaths during 2010–2011. The dataset used for this study was also contributed to the multisite INDEPTH Network cause-specific mortality dataset (14).

### Ethical statement

The KA-HDSS received ethical clearance from the Ethiopian Science and Technology Agency with identification number – IERC 0030. Informed verbal consent was obtained from every respondent. The consent procedure was stated in the proposal which was approved by the ethical review committee.

## Results

VA data were collected for a total of 436/599 (72.7%) deaths that occurred in KA-HDSS during 2010–2011. These were processed by the InterVA-4 model which assigned cause(s) of death to all except 35 cases (8.0%) which were completely indeterminate. Residual components assigned to indeterminate cause amounted to an additional 33.5 (7.7%) of cases. Ninety percent of the deceased were from rural areas and the median age at death was 58 years (inter quartile range = 33 years). Most deaths, about 89%, took place outside health facilities. Neonates accounted for 6.9% of the cases, post neonates for 5.7%, children of 1–4 years for 5.3%, and those 5–14 for 6.9%. Among adults, age groups 15–49, 50–65 years, and 65-plus years accounted for 20.2, 12.2, and 42.9%, respectively.

Overall, 152.8 deaths (35.0%) were attributed to communicable diseases, 133.4 deaths (30.7%) to chronic non-communicable diseases, and 28.3 deaths (6.5%) to neonatal causes (Table 1). Tuberculosis (TB) and acute respiratory tract infections (ARTI) including pneumonia were frequent communicable CoD, contributing 12.5 and 10.4% of the overall mortality, respectively. Neoplasms and diseases of the circulatory system were major chronic non-communicable causes, contributing 9.6 and 7.2% of the deaths, respectively. Neonatal pneumonia (4.0%) and external causes (9.3%) were the other important components of overall mortality.

**Table 1:** InterVA-4 based cause of death by sex in KA-HDSS Ethiopia, 2010–2011

Cause of death	WHO VA code	Female *N* (%)	Male *N* (%)	Total *N* (%)
I. Communicable diseases		75.4 (38.5)	77.4 (32.2)	152.8 (35.0)
Tuberculosis	VA-01.09	25.9 (13.2)	28.7 (12.0)	54.6 (12.5)
ARTI, including pneumonia	VA-01.02	21.9 (11.2)	23.4 (9.7)	45.3 (10.4)
HIV/AIDS related	VA-01.03	8.8 (4.5)	7.8 (3.2)	16.6 (3.8)
Malaria	VA-01.05	6.0 (3.1)	5.9 (2.5)	11.9 (2.7)
Diarrheal diseases	VA-01.04	5.8 (2.9)	3.3 (1.4)	9.0 (2.1)
Others	Other VA-01	7.0 (3.6)	8.3 (3.4)	15.4 (3.5)
II. Non-communicable diseases:		56.8 (29.0)	77.0 (32.1)	133.4 (30.7)
Neoplasms		20.4 (10.4)	21.6 (9.0)	41.9 (9.6)
Respiratory neoplasms	VA-02.03	6.2 (3.2)	9.8 (4.1)	16.0 (3.7)
Other neoplasms	VA-02.99	4.7 (2.4)	5.8 (2.4)	10.5 (2.4)
Digestive neoplasms	VA-02.02	6.9 (3.5)	4.4 (1.8)	11.3 (2.6)
Reproductive neoplasms	VA-02.05, 06	2.6 (1.3)	1.6 (0.7)	4.2 (1.0)
Diseases of the circulatory system		13.1 (6.7)	18.5 (7.7)	31.4 (7.2)
Stroke	VA-04.02	8.1 (4.1)	8.8 (3.7)	16.9 (3.9)
Other cardiac disease	VA-04.99	4.1 (2.1)	6.8 (2.8)	10.9 (2.5)
Acute cardiac disease	VA-04.01	0.9 (0.5)	2.9 (1.2)	3.8 (0.8)
Gastrointestinal disorders	VA-06.01, 02	4.2 (2.1)	17.1 (7.1)	21.3 (4.9)
Diabetes mellitus	VA-03.03	2.7 (1.4)	2.9 (1.2)	5.6 (1.3)
Mental disorders: epilepsy	VA-08.01	3.9 (2.0)	7.3 (3.0)	11.6 (2.6)
Respiratory disorders^a^	VA-05.01, 02	6.6 (3.4)	6.0 (2.5)	12.6 (2.9)
Renal disorders: renal failure	VA-07.01	2.5 (1.3)	2.1 (0.9)	4.5 (1.0)
Other and unspecified NCDs	VA-98	3.4 (1.7)	1.5 (0.6)	4.9 (1.1)
III. Neonatal causes of death		9.6 (4.9)	18.7 (7.8)	28.3 (6.5)
Neonatal pneumonia	VA-10.03	7.0 (3.6)	10.5 (4.4)	17.5 (4.0)
Other neonatal	Other VA-10	2.6 (1.3)	8.2 (3.4)	10.8 (2.5)
IV. External causes of death		14.5 (7.4)	26.1 (10.9)	40.6 (9.3)
Accidental fall, drowning	VA-12.03-04	3.8 (1.9)	8.5 (3.5)	12.3 (2.8)
Self-harm, assault	VA-12.08-09	2.7 (1.4)	9.0 (3.8)	11.7 (2.7)
Road traffic accident	VA-12.01	2.0 (1.0)	4.5 (1.9)	6.5 (1.5)
Others & unspecified	Other VA-12	6.0 (3.1)	4.1 (1.7)	10.1 (2.3)
V. Malnutrition	VA-03.01-02	4.9 (2.5)	5.0 (2.1)	10.0 (2.3)
VI. Maternal causes	VA-09	2.4 (1.2)	**–**	2.4 (0.6)
VII. Indeterminate^b^	VA-99	32.6 (16.6)	35.9 (15.0)	68.5 (15.7)
Total		196 (100.0)	240 (100.0)	436 (100.0)

a Chronic obstructive pulmonary disease, Asthma

b residual and completely indeterminate.

Chronic non-communicable diseases and communicable diseases caused comparable proportions of deaths in both sexes. A large proportion of neonatal deaths (58.3%) was attributed to neonatal pneumonia (Table 2). ARTI including pneumonia was the leading cause of death in infants and children, accounting for 72.0 and 17.0%, respectively. Among adults, TB was the leading cause of death in age groups 15–49, 50–65, and 65-plus years, accounting for 19.9, 24.5, and 12.4%, respectively.

**Table 2:** Leading InterVA-4 based causes of death by age group, KA-HDSS Ethiopia, 2010–2011

	*N* (%)
Neonates (*n*=30)	
Neonatal pneumonia	17.5 (58.3)
Unspecified neonatal CoD	5.5 (18.3)
Birth asphyxia	2.9 (9.6)
Infants (*n*=25)	
ARTI, including pneumonia	18.0 (72.0)
Indeterminate	2.7 (10.8)
HIV/AIDS related	1.9 (7.6)
1–4 years (*n*=23)	
Indeterminate	5.3 (23.0)
ARTI, including pneumonia	3.9 (17.0)
HIV/AIDS related	2.9 (12.6)
5–14 years (*n*=30)	
Indeterminate	6.0 (20.0)
Accidental drowning	3.0 (10.0)
Road traffic accident	2.5 (8.3)
15–49 years (*n*=88)	
Tuberculosis	17.5 (19.9)
Indeterminate	7.7 (8.8)
Acute abdomen	5.0 (5.7)
50–64 years (*n*=53)	
Tuberculosis	13.0 (24.5)
Indeterminate	8.7 (16.4)
HIV/AIDS related	4.1 (7.7)
65-plus years (*n*=187)	
Indeterminate	37.4 (20.0)
Tuberculosis	23.1 (12.4)
ARTI, including pneumonia	14.6 (7.8)

## Discussion

In this rural community, where reliable sources of CoD data are absent, the InterVA model generated plausible estimates of the major public health problems. Moreover, InterVA yields CoD which is completely internally consistent, allowing comparisons of data from different countries. It is also less labor intensive as compared to physician review. Despite its computational simplicity, it is certainly true that using any mathematical model for interpreting cause of death may not reflect the subjective subtleties of physician review, barring inconsistent physician reviews.

The proportion of deaths attributed to chronic non-communicable causes in our study (30.7%) was similar to the 34.5% (cardiac diseases, other non-communicable diseases, diabetes) reported in a similar study from rural north western Ethiopia (15). Comparable estimates were also reported from other studies in Ethiopia that used hospital records (31.0%) and physician review (28.6%) methods (16, 17). The preponderance of chronic non-communicable causes in the rural setting is likely to be explained by rapid socioeconomic development and parallel large-scale investments in health care (12, 18). According to the World Bank, the Ethiopian economy has experienced strong and broad-based growth over the past decade, averaging 9.9% per year in 2004–05 to 2011–12 compared to the East African average of 5.4% (19). As a result, there is an increase in life expectancy (12), as well as exposure to risk factors for chronic non-communicable diseases (20). A recent survey in rural south western Ethiopia showed that 80% of the population surveyed had at least one risk factor for chronic non-communicable diseases (20). Several studies from Ethiopia also showed that chronic non-communicable diseases are increasingly becoming more apparent health problems (20–22).

The contribution of communicable causes to the overall deaths in our study (35.0%) was lower than the 47.5% (TB, HIV/AIDS, and other infectious disease) reported from northwestern Ethiopia (15) and much lower than the 58.0% in Kenya (2). Misganaw et al. also reported that mortality from communicable, maternal, neonatal, and nutritional conditions have decreased from 68.0% in 2002 to 41.0% in 2010 (16). Despite the variation in the estimates, both studies showed that the burden of communicable diseases in Ethiopia has declined. This could also be explained by the improvements in health and socioeconomic status of the population. Primary health service coverage has now reached 92% (23). The national health care program, which focuses on health promotion and prevention of common health problems also, is likely to have played a significant role (12). Deaths from malaria have declined by 50% between 2007–8 and 2011, child mortality rate by 28.4%, during 2005–2010, and HIV/AIDS prevalence among the adults has dropped to 1.5% in 2010–11 (12).

In our study, TB and ARTI including pneumonia were frequently diagnosed communicable CoD. TB was also identified as the leading communicable CoD in other similar studies, but the estimates attributed to TB were higher than in our findings (12.5%); 36% in Ethiopia and 31% in Nairobi, Kenya (2, 15). A lower prevalence of TB than the national estimate was also reported in a recent survey in the region where our study was conducted (24). Studies from Ethiopia reported comparable mortality estimates attributed to ARTI including pneumonia (16, 17). The proportion of deaths attributed to HIV/AIDS in our study (3.8%) was much lower than findings from other studies in Ethiopia (7.6%), Nairobi (17.0%), and Kilifi (12.4%) in Kenya (2, 10, 15). Despite, the geographic variation in the prevalence of HIV/AIDS, in three of these studies (2, 10, 15), the level of HIV was set to be ‘high’ in the model which might have affected the estimates.

Diseases arising during the neonatal period were important CoD next to the two leading groups of CoD. This was comparable to findings from rural south Ethiopia, where 6.5% of total deaths were attributed to neonatal causes and premature deaths (16). In our findings, neonatal pneumonia was the major killer among neonates, causing more than half of all deaths during the neonatal period. Generally, pneumonia is the major cause of neonatal deaths in developing countries (25).

The model also estimated deaths from accidents and injuries consistent to findings from Ethiopia (9.6%), Kenya (8.8%), and the global WHO estimate (9%) (2, 15, 26). In our study, deaths from accidents and injuries were more prevalent in males, and children aged 5–14 were more affected than the other age groups. This was consistent with findings from Uganda and a WHO global report on injuries (26). The sex difference in burden of deaths from accidents and injuries is explained by variation in the roles men have in most societies. Males often engage in more hazardous and risky jobs than females (26). Children are also more vulnerable to accidents and injuries as they are less able to predict and prevent accidents than adults (26).

This study used standardized data collection tools and trained full-time data collectors. Moreover, the VA data analyzed in this study were collected as part of the routine follow-up of the KA-HDSS, which would have minimized recall bias. However, this study will have limitations inherent to limitations of the VA process.

## Conclusion

In general, the major public health problems identified by the InterVA model were comparable to the expected local burden of diseases. Communicable diseases and chronic non-communicable diseases caused similar proportions of deaths. Neoplasms and diseases of the circulatory system were the major chronic non-communicable causes. TB and acute respiratory infections were the leading specific CoD. In countries where death certification is non-existent, the InterVA tool is feasible for generating cause of death data that would be satisfactory to guide public health interventions. We encourage validation studies, in local settings, so that the InterVA can be integrated into the national health surveys to yield nationwide cause of death data.

## References

[1] Vergnano S, Fottrell E, Osrin D, Kazembe PN, Mwansambo C, Manandhar DS (2011). Adaptation of a probabilistic method (InterVA) of verbal autopsy to improve the interpretation of cause of stillbirth and neonatal death in Malawi, Nepal, and Zimbabwe. Popul Health Metr.

[2] Oti S, Kyobutungi C (2010). Verbal autopsy interpretation: a comparative analysis of the InterVA model versus physician review in determining causes of death in the Nairobi DSS. Popul Health Metr.

[3] Byass P (2001). Person, place and time – but who, where, and when?. Scand J Public Health.

[4] King G, Ying Lu Y (2008). Verbal autopsy methods with multiple causes of death. Stat Sci.

[5] Setel PW, Sankoh O, Rao C, Velkoff VA, Mathers C, Gonguan Y (2005). Sample registration of vital events with verbal autopsy: a renewed commitment to measuring and monitoring vital statistics. Bull World Health Organ.

[6] King G, Lu Y, Shibuya K (2010). Designing verbal autopsy studies. Popul Health Metr.

[7] Byass P, Chandramohan D, Clark SJ, D'Ambruoso L, Fottrell E, Graham WJ (2012). Strengthening standardized interpretation of verbal autopsy data: the new InterVA-4 tool. Glob Health Action.

[8] World Health Organization Verbal autopsy standards: the 2012 WHO verbal autopsy instrument. http://www.who.int/healthinfo/statistics/WHO_VA_2012_RC1_Instrument.pdf.

[9] SAVVY Sample vital registration with verbal autopsy. http://www.cpc.unc.edu/measure/tools/monitoring-evaluation-systems/savvy.

[10] Bauni E, Ndila C, Mochamah G, Nyutu G, Matata L, Ondieki C (2011). Validating physician-certified verbal autopsy and probabilistic modeling (InterVA) approaches to verbal autopsy interpretation using hospital causes of adult deaths. Popul Health Metr.

[11] Fantahun M, Fottrell E, Berhane Y, Wall S, Hogberg U, Byass P (2006). Assessing a new approach to verbal autopsy interpretation in a rural Ethiopian community: the InterVA model. Bull World Health Organ.

[12] Federal Democratic Republic of Ethiopia Ministry of Finance and Economic Development (2012) MDGs Report 2012 Addis Ababa, Ethiopia. http://www.mofed.gov.et/English/Resources/Documents/Ethiopia_MDG_Report_2012.pdf.

[13] Central Statistical Agency [Ethiopia] and ICF International (2012). Ethiopia demographic and health survey 2011.

[14] INDEPTH Network INDEPTH Network Cause-Specific Mortality - Release 2014. Oct 2014. Provided by the INDEPTH Network Data Repository. http://www.indepth-network.org.

[15] Tadesse S (2013). Validating the InterVA model to estimate the burden of mortality from verbal autopsy data: a population-based cross-sectional study. PLoS One.

[16] Misganaw A, Haile Mariam D, Araya T, Ayele K (2012). Patterns of mortality in public and private hospitals of Addis Ababa. Ethiopia. BMC Public Health.

[17] Weldearegawi B, Ashebir Y, Gebeye E, Gebregziabiher T, Yohannes M, Mussa S (2013). Emerging chronic non-communicable diseases in rural communities of Northern Ethiopia: evidence using population-based verbal autopsy method in Kilite Awlaelo surveillance site. Health Policy and Plann.

[18] Federal Democratic Republic of Ethiopia, Ministry of Health (2010). Health Sector Development Program IV.

[19] World Bank (2013). Global Indicators. http://data.worldbank.org/topic/health.

[20] Alemseged F, Haileamlak A, Tegegn A, Tessema F, Woldemichael K, Asefa M (2012). Risk factors for chronic non-communicable diseases at Gilgel gibe field research center, southwest Ethiopia: population based study. Ethiop J Health Sci.

[21] Prevett M (2012). Chronic non-communicable diseases in Ethiopia a hidden burden. Ethiop J Health Sci.

[22] Mamo Y, Seid E, Adams SS, Gardiner A, Parry E (2007). A primary healthcare approach to the management of chronic disease in Ethiopia: an example for other countries. Clin Med.

[23] Federal Democratic Republic of Ethiopia, Ministry of Health (2011). Health and health related indicators.

[24] 
Berhe G, Enqueselassie F, Hailu E, Mekonnen W, Teklu T, Gebretsadik A (2013). Population-based prevalence survey of tuberculosis in the Tigray region of Ethiopia. BMC Infect Diss.

[25] World Health Organization (2013). Children: reducing child mortality. http://www.who.int/mediacentre/factsheets/fs178/en/.

[26] World Health Organization (2008). UNICEF. World report on child injury prevention.

